# With expanded carrier screening, founder populations run the risk of being overlooked

**DOI:** 10.1007/s12687-017-0309-5

**Published:** 2017-05-29

**Authors:** Inge B. Mathijssen, Merel C. van Maarle, Iris I.M. Kleiss, Egbert J.W. Redeker, Leo P. ten Kate, Lidewij Henneman, Hanne Meijers-Heijboer

**Affiliations:** 1 Department of Clinical Genetics, Academic Medical Center, Amsterdam, The Netherlands; 2 Department of Clinical Genetics, VU University Medical Center, Amsterdam, The Netherlands; 3 Amsterdam Public Health Research Institute, VU University Medical Center, Amsterdam, The Netherlands

**Keywords:** Founder population, Community, Carrier screening, Autosomal recessive, Netherlands

## Abstract

Genetically isolated populations exist worldwide. Specific genetic disorders, including rare autosomal recessive disorders may have high prevalences in these populations. We searched for Dutch genetically isolated populations and their autosomal recessive founder mutations. We investigated whether these founder mutations are covered in the (preconception) expanded carrier screening tests of five carrier screening providers. Our results show that the great majority of founder mutations are not covered in these screening panels, and these panels may thus not be appropriate for use in founder populations. It is therefore important to be aware of founder mutations in a population when offering carrier tests.

## Introduction

The general Dutch population is relatively outbred (Ten Kate et al. 2014). However, several genetically isolated populations exist (for definitions of terms see Box 1). Also, in many other countries worldwide, genetically isolated populations are present. Often these populations are geographically, culturally, or for religious reasons isolated for centuries and as a consequence show less genetic variation. Because of the low genetic heterogeneity, these populations have proven to be very suitable for the identifications of genes involved both in Mendelian as well as in complex disorders. Bottleneck effects in the history of a population and/or founder effects may result in high prevalences of monogenic recessive disorders in these populations compared with the nationwide prevalences.

**Box 1:** Definitions

Genetically isolated population or founder population: a population that is or was geographically, culturally, or for religious reasons isolated and as a consequence has restricted genetic variation. Bottleneck effect: occurs when there is a sharp reduction in the size of a population due to a natural disaster or similar event with, as a consequence, reduction of the genetic variation in the population. Founder effect: the reduction of genetic variation that occurs when a new population is founded by a small number of individuals (founders) from a larger population and this population remains isolated to other populations. Founder mutation: a gene mutation on an identical haplotype background, observed with high frequency in a genetically isolated population in which one or more of the ancestors were carriers of the gene mutation. Recurrent mutation: a gene mutation on more than one haplotype background, reoccurring multiple times in a population history.	

Because of high carrier frequencies in founder populations, carrier screening programs have been introduced in some of these populations. The aim of these programs is to identify carrier couples with a one-in-four risk of affected offspring, enabling autonomous reproductive decision making, which consequently might reduce perinatal morbidity and mortality. Examples are carrier screening programs for genetic disorders in people of Eastern European Jewish (Ashkenazi) descent (for example Tay-Sachs disease) (ACOG Committee on Genetics 2009), targeted carrier screening for severe and frequent disorders in different isolated (mainly Arab and Druze) communities in Israel (Basel-Vanagaite et al. 2007; Falik-Zaccai et al. 2008; Zlotogora et al. 2009), screening for four recessive diseases in the Saguenay-Lac-Saint-Jean region of Quebec, Canada (Tardif et al. 2017), and screening for four severe autosomal recessive disorders in a Dutch genetically isolated community (Mathijssen et al. 2015).

Meanwhile, technological advances have enabled the development and offer of preconception expanded carrier screening (ECS) in which couples without an a priori increased risk of having a child with a genetic disorder can be screened for several (hundreds of) disorders simultaneously (Edwards et al. 2015; Henneman et al. 2016). An increasing number of mainly commercial laboratories offer these screening panels (Borry et al. 2011; Lazarin et al. 2013).

However, the carrier frequencies of several autosomal recessive disorders in genetically isolated populations can be very skewed from nationwide or worldwide carrier frequencies. In some genetically isolated populations, carrier frequencies of disorders which are rare or almost non-existent in the general population may be very high. In contrast, carrier frequencies of more frequent disorders in the general population may be very low in genetically isolated populations.

The aim of this study was to make an inventory of Dutch genetically isolated populations and their autosomal recessive founder mutations, and to investigate whether Dutch founder mutations are covered in the (preconception) expanded carrier screening tests of carrier screening providers.

## Methods

We searched for genetically isolated populations in the Netherlands (total population 17 million people) and their founder mutations in the databases PubMed, On-line Mendelian Inheritance in Man (OMIM), and Google semi-systematically by using the keywords “genetically isolated population,” “founder,” “mutation,” “gene,” and “Dutch” or “Netherlands.” Also, 11 Dutch clinical (molecular) geneticists were asked about their knowledge of genetically isolated populations and their specific founder mutations. Only autosomal recessive mutations were included. Recurrent mutations (Box 1) and Dutch founder mutations not related to a specific genetically isolated community were not included (Zeegers et al. 2004). To prevent possible stigmatization, the genetically isolated populations are numbered and the specific names of the villages are not mentioned.

Our purpose was not to be complete, but to illustrate the importance of being aware of founder populations and founder mutations. We therefore made a selection of founder mutations present in different genetically isolated populations for which the most information was available in the literature and from personal information.

Information about the carrier frequencies of founder mutations were derived from the scientific articles and personal communication with clinical (molecular) geneticists. Carrier frequencies in the Dutch general population were derived from The Genome of the Netherlands (GoNL) project (http://www.nlgenome.nl, accessed 24 February 2017) (The Genome of the Netherlands Consortium 2014).

We investigated whether the specific founder mutations were present or absent in the expanded carrier screening panels offered by five ECS providers.

## Results

In Table 1, several founder mutations present in six different Dutch genetically isolated populations are shown, including the carrier frequencies of these disorders in the specific genetically isolated population and in the Dutch general population. As can be seen, the carrier frequencies of generally rare disorders are high in these genetically isolated populations.

**Table 1:** Founder mutations in different Dutch genetically isolated communities and the coverage of these mutations in expanded carrier screening tests

Disorder	OMIM	Disease severity^a^	Gene	Mutation	Population^b^	Carrier frequency genetic isolate^c^	Carrier frequency Dutch general population^d^	Counsyl^e^	GenPath Diagnostics^f^	Mount Sinai^g^	Pathway Genomics^h^	Recombine^i^	References
Fetal akinesia deformation sequence (FADS)	208150	4	*MUSK*	c.1724T>C p.(Ile575Thr)	1	8.1–11.2%	<0.2%	−	−	−	−	−	Mathijssen et al. (2015) and Tan-Sindhunata et al. (2015)
Osteogenesis imperfecta type IIB/III (OI)	610682	3	*CRTAP*	c.21_22dupGG p.(Ala8fs)	1	4.1%	<0.2%	−	−	−	−	−	Mathijssen et al. (2015) and van Dijk et al. (2009)
Phenylketonuria (PKU)	261600	3	*PAH*	c.1315+1G>A	1	9.2%	<0.2%	+	+	+	–^j^	**+**	Oorthuys et al. (1985)
Pontocerebellar hypoplasia type 2 (PCH2)	277470	4	*TSEN54*	c.919G>T p.(Ala307Ser)	1	1.5–14.3%	0.8%	−	−	−	−	**+**	Barth et al. (1990), Budde et al. (2008), and Mathijssen et al. (2015)
Primary ciliary dyskinesia (PCD)	615067	2	*CCDC114*	c.742G>A p.(Gly248Thrfs)	1	10%	0.4%	−	−	−	−	−	Onoufriadis et al. (2013)
Pseudoxanthoma elasticum (PXE)	264800	3	*ABCC6*	c.3775delT p.(Trp1259Glyfs)	1	15 cases described (~21,500)	0.8%	−	–^j^	−	−	−	Bergen et al. (2000) and Plomp et al. (2009)
Retinitis pigmentosa type 12 (RP12)	600105	2	*CRB1*	c.3122T>C p.(Met1041Thr)	1	4.1%	<0.2%	−	–^j^	**+**	−	−	Den Hollander et al. (1999) and Mathijssen et al. (2017)
Rhizomelic chondrodysplasia punctata type 1 (RCDP1)	215100	4	*PEX7*	c.875T>A p.(Leu292X)	1	6.1%	<0.2%	**+**	**+**	**+**	−	**+**	Mathijssen et al. (2015)
Van Buchem disease (VBCH)	239100	3	*SOST*	52 kb deletion approximately 35 kb downstream of gene	2	≥13 cases described (~20,000)	<0.2%	−	−	−	−	−	Balemans et al. (2002) and Van Buchem et al. (1955)
Congenital retinal dystrophy	204100	2	*RPE65*	c.1102T>C p.(Tyr368His)	2	3.1–7.7%	0.4%	−	−	**+**	−	**+**	Schappert-Kimmijser et al. (1959) and Yzer et al. (2003)
Retinitis punctata albescens (RPA)	136880	2	*LRAT*	c.12delC p.(Met5CysfsX53)	2	4 cases described (~20,000)	<0.2%	−	−	−	−	−	Littink et al. (2012)
Vici syndrome (variant form)	242840	4	*EPG5*	c.4862G>A p.(Arg1621Gln)	2	±10% (pc)	<0.2%	−	−	−	−	−	
Benign recurrent intrahepatic cholestasis (BRIC)	243300	2	*ATP8B1*	c.2932-3C>A, skipping of exon 24	3	≥7 cases described (~20,000)	<0.2%	−	−	−	−	−	De Koning et al. (1995), Houwen et al. (1994) and Klomp et al. (2004)
Chudley-McCullough syndrome (CMCS)	604213	2	*GPSM2*	c.1473delG p.(Phe492SerfsX5)	4	3 cases described (~15,000)	<0.2%	−	−	−	−	−	Almomani et al. (2013) and Hendriks et al. (1999)
Juvenile neuronal ceroid lipofuscinosis (CLN3)	204200	4	*CLN3*	1.02 kb deletion	5	17 cases described	0.5%	**+**	−	**+**	−	−	Taschner et al. (1995)
Parkinson disease type 7 (PARK7)	606324	3	*DJ-1*	14 kb deletion	6	0.9%	<0.2%	−	−	−	−	−	Bonifati et al. (2003)

“+” presence of the mutation in panel. “−” absence of the mutation in panel, *pc* personal communications

^a^Disease severity; 1 = mild, 2 = moderate, 3 = severe, 4 = profound (Lazarin et al. 2014). Scored independently by IM and IK. Discrepancies were discussed until consensus was reached

^b^Six different Dutch founder populations (coded from 1 to 6)

^c^If the carrier frequency in the genetically isolated community is not known, the number of cases described in the literature and the current population size of the genetically isolated community is noted

^d^Derived from Genome of the Netherlands (GoNL) project, http://www.nlgenome.nl; accessed 23 February 2017

^e^Family Prep Screen (113 disorders); https://www.counsyl.com/services/family-prep-screen; accessed 6 February 2017

^f^InheriGen Plus (167 disorders); http://www.genpathdiagnostics.com/womens-health/inherigen; accessed 6 February 2017

^g^NextStep Pan-Ethnic Carrier Screen (281 disorders); http://nextsteptest.com; accessed 6 February 2017

^h^Carrier Status DNA Insight (72 disorders); https://www.pathway.com/carrier-status-dna-insight; accessed 6 February 2017

^i^CarrierMap (315 disorders); http://www.recombine.com/carriermap; accessed 6 February 2017

^j^A selection of mutations is included in the carrier screening, but the founder mutation is not

For the five selected carrier screening providers, the coverage of 16 founder mutations in the carrier screening tests is shown. For each carrier screening provider, on average 2.8 (range 0–5) of the 16 founder mutations are covered in the test. Eleven (69%) of the founder mutations are covered in none of the five carrier screening tests. In the test of two providers, a selection of mutations in the specific gene of three disorders is included in the carrier screening test, but the founder mutation is not.

## Discussion

In genetically isolated populations, carrier frequencies of genetic disorders can be very different from the carrier frequencies in the general population. The great majority of these founder mutations are not covered in the ECS panels of the five selected providers. This also applies to most of the founder mutations present in genetically isolated populations in other countries.

Offering a (commercial) routine ECS panel to inhabitants of these genetically isolated populations is not appropriate because it may give false reassurance to couples with an increased risk for founder mutations related disorders they are not being tested for.

For a reliable carrier test offer, it is therefore important to know the genetically isolated populations and their founder mutations in each country. Nationwide databases in which the genetically isolated populations, their relatively frequent genetic disorders, and the specific genes and mutations are listed, are a suitable solution. A database is not only very important for carrier screening programs but also for making a rapid (differential) diagnosis by clinicians, genetic counseling, and research in these populations. For some genetically isolated populations such (online) databases are already available. Examples are the Amish, Mennonite, and Hutterite Genetic Disorder Database (www.biochemgenetics.ca/plainpeople) (Payne et al. 2011) and the Israeli National Genetic Database (www.goldenhelix.org/israeli) (Zlotogora 2010).

Ideally, a customized carrier test will be developed for each country/region in which both country/region-specific mutations as well as genetic isolate-specific founder mutations are present. This approach may also reduce potential stigmatization of genetic isolates, while specific mutations still are included.

In the Netherlands, carrier screening is only (partly) paid by health insurance companies in case of an increased risk of being a carrier; e.g. carrier screening for four autosomal recessive disorders in a genetically isolated community (Mathijssen et al. 2015) and carrier screening for nine disorders in individuals of Ashkenazi Jewish descent (Holtkamp et al. 2016). Recently, the Academic Medical Center in Amsterdam started a non-profit offer of carrier screening for 50 severe autosomal recessive disorders. Most of the severe disorders currently known in Dutch genetically isolated populations are included. Probably, in a non-commercial setting, it will be more likely to take into account the founder mutations present in founder populations than for commercial companies to include those mutations.

It is expected that in the near future, it will become possible to use whole-exome sequencing (WES) or whole-genome sequencing (WGS) for ECS, in which all known disease genes can be screened, including very rare disease genes prevalent in genetically isolated populations. However, correct interpretation of test-results (e.g. variants of unknown clinical significance) when using WES or WGS is complex. Also, the identification of carrier couples for mild disorders which are unlikely to alter reproductive plans is an important point to take into consideration (Beaudet 2015; Sallevelt et al. 2016).

In the meantime, descendants from genetically isolated populations should be made aware that a (commercial) routine ECS panel may not be appropriate and can give false reassurance because the population-specific genes and/or mutations are not covered. Currently, this information is not mentioned by expanded carrier test providers.

Not only genetically isolated populations but also consanguineous couples and populations with a high rate of consanguineous unions have an increased risk of having a child with an autosomal recessive disorder (Teeuw et al. 2014). Many of these disorders are rare disorders which are also not covered in the (commercial) routine ECS panels. These panels may therefore also not be appropriate for use in consanguineous couples and in countries/populations with a high rate of consanguineous unions.

## Conclusions

In genetically isolated populations, carrier frequencies of generally rare autosomal recessive founder mutations can be very high. The great majority of these founder mutations are not covered in most (commercial) routine ECS panels. It is important to be aware of founder populations and founder mutations when using these ECS panels and to check whether the mutations are covered. If these founder mutations are not covered, customized screening tests should be developed which include the founder mutations.

## References

[CR1] ACOG Committee on Genetics (2009). ACOG Committee opinion no. 442: preconception and prenatal carrier screening for genetic diseases in individuals of Eastern European Jewish descent. Obstet Gynecol.

[CR2] Almomani R, Sun Y, Aten E, Hilhorst-Hofstee Y, Peeters-Scholte CM, van Haeringen A, Hendriks YM, den Dunnen JT, Breuning MH, Kriek M, Santen GW (2013). GPSM2 and Chudley-McCullough syndrome: a Dutch founder variant brought to North America. Am J Med Genet A.

[CR3] Balemans W, Patel N, Ebeling M, van Hul E, Wuyts W, Lacza C, Dioszegi M, Dikkers FG, Hildering P, Willems PJ, Verheij JB, Lindpaintner K, Vickery B, Foernzler D, van Hul W (2002). Identification of a 52 kb deletion downstream of the SOST gene in patients with van Buchem disease. J Med Genet.

[CR4] Barth PG, Vrensen GF, Uylings HB, Oorthuys JW, Stam FC (1990). Inherited syndrome of microcephaly, dyskinesia and pontocerebellar hypoplasia: a systemic atrophy with early onset. J Neurol Sci.

[CR5] Basel-Vanagaite L, Taub E, Halpern GJ, Drasinover V, Magal N, Davidov B, Zlotogora J, Shohat M (2007). Genetic screening for autosomal recessive nonsyndromic mental retardation in an isolated population in Israel. Eur J Hum Genet.

[CR6] Beaudet AL (2015). Global genetic carrier testing: a vision for the future. Genome Med.

[CR7] Bergen AA, Plomp AS, Schuurman EJ, Terry S, Breuning M, Dauwerse H, Swart J, Kool M, van Soest S, Baas F, ten Brink JB, de Jong PT (2000). Mutations in ABCC6 cause pseudoxanthoma elasticum. Nat Genet.

[CR8] Bonifati V, Rizzu P, van Baren MJ, Schaap O, Breedveld GJ, Krieger E, Dekker MC, Squitieri F, Ibanez P, Joosse M, van Dongen JW, Vanacore N, van Swieten JC, Brice A, Meco G, van Duijn CM, Oostra BA, Heutink P (2003). Mutations in the DJ-1 gene associated with autosomal recessive early-onset parkinsonism. Science.

[CR9] Borry P, Henneman L, Lakeman P, ten Kate LP, Cornel MC, Howard HC (2011). Preconceptional genetic carrier testing and the commercial offer directly-to-consumers. Hum Reprod.

[CR10] Budde BS, Namavar Y, Barth PG, Poll-The BT, Nurnberg G, Becker C, van Ruissen F, Weterman MA, Fluiter K, te Beek ET, Aronica E, van der Knaap MS, Hohne W, Toliat MR, Crow YJ, Steinling M, Voit T, Roelenso F, Brussel W, Brockmann K, Kyllerman M, Boltshauser E, Hammersen G, Willemsen M, Basel-Vanagaite L, Krageloh-Mann I, de Vries LS, Sztriha L, Muntoni F, Ferrie CD, Battini R, Hennekam RC, Grillo E, Beemer FA, Stoets LM, Wollnik B, Nurnberg P, Baas F (2008). tRNA splicing endonuclease mutations cause pontocerebellar hypoplasia. Nat Genet.

[CR11] De Koning TJ, Sandkuijl LA, de Schryver JE, Hennekam EA, Beemer FA, Houwen RH (1995). Autosomal-recessive inheritance of benign recurrent intrahepatic cholestasis. Am J Med Genet.

[CR12] Den Hollander AI, ten Brink JB, de Kok YJ, van Soest S, van den Born LI, van Driel MA, van de Pol DJ, Payne AM, Bhattacharya SS, Kellner U, Hoyng CB, Westerveld A, Brunner HG, Bleeker-Wagemakers EM, Deutman AF, Heckenlively JR, Cremers FP, Bergen AA (1999). Mutations in a human homologue of Drosophila crumbs cause retinitis pigmentosa (RP12). Nat Genet.

[CR13] Edwards JG, Feldman G, Goldberg J, Gregg AR, Norton ME, Rose NC, Schneider A, Stoll K, Wapner R, Watson MS (2015). Expanded carrier screening in reproductive medicine-points to consider: a joint statement of the American College of Medical Genetics and Genomics, American College of Obstetricians and Gynecologists, National Society of Genetic Counselors, Perinatal Quality Foundation, and Society for Maternal-Fetal Medicine. Obstet Gynecol.

[CR14] Falik-Zaccai TC, Kfir N, Frenkel P, Cohen C, Tanus M, Mandel H, Shihab S, Morkos S, Aaref S, Summar ML, Khayat M (2008). Population screening in a Druze community: the challenge and the reward. Genet Med.

[CR15] Hendriks YM, Laan LA, Vielvoye GJ, van Haeringen A (1999). Bilateral sensorineural deafness, partial agenesis of the corpus callosum, and arachnoid cysts in two sisters. Am J Med Genet.

[CR16] Henneman L, Borry P, Chokoshvili D, Cornel MC, van El CG, Forzano F, Hall A, Howard HC, Janssens S, Kayserili H, Lakeman P, Lucassen A, Metcalfe SA, Vidmar L, de Wert G, Dondorp WJ, Peterlin B (2016). Responsible implementation of expanded carrier screening. Eur J Hum Genet.

[CR17] Holtkamp KC, van Maarle MC, Schouten MJ, Dondorp WJ, Lakeman P, Henneman L (2016) Do people from the Jewish community prefer ancestry-based or pan-ethnic expanded carrier screening? Eur J Hum Genet 24:171–177 10.1038/ejhg.2015.97 PMC4717216 25966636

[CR18] Houwen RH, Baharloo S, Blankenship K, Raeymaekers P, Juyn J, Sandkuijl LA, Freimer NB (1994). Genome screening by searching for shared segments: mapping a gene for benign recurrent intrahepatic cholestasis. Nat Genet.

[CR19] Klomp LW, Vargas JC, van Mil SW, Pawlikowska L, Strautnieks SS, van Eijk MJ, Juijn JA, Pabon-Pena C, Smith LB, DeYoung JA, Byrne JA, Gombert J, van der Brugge G, Berger R, Jankowska I, Pawlowska J, Villa E, Knisely AS, Thompson RJ, Freimer NB, Houwen RH, Bull LN (2004). Characterization of mutations in ATP8B1 associated with hereditary cholestasis. Hepatology.

[CR20] Lazarin GA, Haque IS, Nazareth S, Iori K, Patterson AS, Jacobson JL, Marshall JR, Seltzer WK, Patrizio P, Evans EA, Srinivasan BS (2013). An empirical estimate of carrier frequencies for 400+ causal Mendelian variants: results from an ethnically diverse clinical sample of 23,453 individuals. Genet Med.

[CR21] Lazarin GA, Hawthorne F, Collins NS, Platt EA, Evans EA, Haque IS (2014). Systematic classification of disease severity for evaluation of expanded carrier screening panels. PLoS One.

[CR22] Littink KW, van Genderen MM, van Schooneveld MJ, Visser L, Riemslag FC, Keunen JE, Bakker B, Zonneveld MN, den Hollander AI, Cremers FP, van den Born LI (2012). A homozygous frameshift mutation in LRAT causes retinitis punctata albescens. Ophthalmology.

[CR23] Mathijssen IB, Florijn RJ, van den Born LI, Zekveld-Vroon RC, ten Brink JB, Plomp AS, Baas F, Meijers-Heijboer H, Bergen AA, van Schooneveld MJ (2017). Long-term follow-up of patients with retinitis pigmentosa type 12 caused by CRB1-mutations: a severe phenotype with considerable Interindividual variability. Retina.

[CR24] Mathijssen IB, Henneman L, van Eeten-Nijman JM, Lakeman P, Ottenheim CP, Redeker EJ, Ottenhof W, Meijers-Heijboer H, van Maarle MC (2015). Targeted carrier screening for four recessive disorders: high detection rate within a founder population. Eur J Med Genet.

[CR25] Onoufriadis A, Paff T, Antony D, Shoemark A, Micha D, Kuyt B, Schmidts M, Petridi S, Dankert-Roelse JE, Haarman EG, Daniels JM, Emes RD, Wilson R, Hogg C, Scambler PJ, Chung EM, Pals G, Mitchison HM (2013). Splice-site mutations in the axonemal outer dynein arm docking complex gene CCDC114 cause primary ciliary dyskinesia. Am J Hum Genet.

[CR26] Oorthuys JW, Gons MH, Kwakman JV, Schutgens RB, Tegelaers WH (1985). Screening for raised serum phenylalanine levels in women of reproductive age in Volendam. Ned Tijdschr Geneeskd.

[CR27] Payne M, Rupar CA, Siu GM, Siu VM (2011). Amish, mennonite, and hutterite genetic disorder database. Paediatr Child Health.

[CR28] Plomp AS, Bergen AA, Florijn RJ, Terry SF, Toonstra J, van Dijk MR, de Jong PT (2009). Pseudoxanthoma elasticum: wide phenotypic variation in homozygotes and no signs in heterozygotes for the c.3775delT mutation in ABCC6. Genet Med.

[CR29] Sallevelt SC, de Koning B, Szklarczyk R, Paulussen AD, de Die-Smulders CE, Smeets HJ (2016) A comprehensive strategy for exome-based preconception carrier screening. Genet Med. doi: 10.1038/gim.2016.153 10.1038/gim.2016.153 28492530

[CR30] Schappert-Kimmijser J, Henkes HE, van den Bosch J (1959) Amaurosis congenita (Leber). AMA Arch Ophthalmol 61:211–218 10.1001/archopht.1959.00940090213003 13616783

[CR31] Tan-Sindhunata MB, Mathijssen IB, Smit M, Baas F, de Vries JI, van der Voorn JP, Kluijt I, Hagen MA, Blom EW, Sistermans E, Meijers-Heijboer H, Waisfisz Q, Weiss MM, Groffen AJ (2015). Identification of a Dutch founder mutation in MUSK causing fetal akinesia deformation sequence. Eur J Hum Genet.

[CR32] Tardif J, Pratte A, Laberge AM (2017) Experience of carrier couples identified through a population-based carrier screening pilot program for four founder autosomal recessive diseases in Saguenay-Lac-Saint-Jean. Prenat Diagn. doi: 10.1002/pd.5055 10.1002/pd.5055 28419508

[CR33] Taschner PE, de Vos N, Post JG, Meijers-Heijboer EJ, Hofman I, Loonen MC, Pinckers AJ, Bleeker-Wagemakers EM, Gardiner RM, Breuning MH (1995). Carrier detection of batten disease (juvenile neuronal ceroid-lipofuscinosis). Am J Med Genet.

[CR34] Teeuw M, Waisfisz Q, Zwijnenburg PJ, Sistermans EA, Weiss MM, Henneman L, Ten Kate LP, Cornel MC, Meijers-Heijboer H (2014). First steps in exploring prospective exome sequencing of consanguineous couples. Eur J Med Genet.

[CR35] Ten Kate LP, Teeuw ME, Henneman L, Cornel MC (2014). Consanguinity and endogamy in the Netherlands: demographic and medical genetic aspects. Hum Hered.

[CR36] The Genome of the Netherlands Consortium (2014). Whole-genome sequence variation, population structure and demographic history of the Dutch population. Nat Genet.

[CR37] Van Buchem FS, Hadders HN, Ubbens R (1955). An uncommon familial systemic disease of the skeleton: hyperostosis corticalis generalisata familiaris. Acta Radiol.

[CR38] Van Dijk FS, Nesbitt IM, Nikkels PG, Dalton A, Bongers EM, van de Kamp JM, Hilhorst-Hofstee Y, den Hollander NS, Lachmeijer AM, Marcelis CL, Tan-Sindhunata GM, van Rijn RR, Meijers-Heijboer H, Cobben JM, Pals G (2009). CRTAP mutations in lethal and severe osteogenesis imperfecta: the importance of combining biochemical and molecular genetic analysis. Eur J Hum Genet.

[CR39] Yzer S, van den Born LI, Schuil J, Kroes HY, van Genderen MM, Boonstra FN, van den Helm B, Brunner HG, Koenekoop RK, Cremers FP (2003). A Tyr368His RPE65 founder mutation is associated with variable expression and progression of early onset retinal dystrophy in 10 families of a genetically isolated population. J Med Genet.

[CR40] Zeegers MP, van Poppel F, Vlietinck R, Spruijt L, Ostrer H (2004). Founder mutations among the Dutch. Eur J Hum Genet.

[CR41] Zlotogora J (2010). The molecular basis of autosomal recessive diseases among the Arabs and Druze in Israel. Hum Genet.

[CR42] Zlotogora J, Carmi R, Lev B, Shalev SA (2009). A targeted population carrier screening program for severe and frequent genetic diseases in Israel. Eur J Hum Genet.

